# Antibody Inhibition of a Viral Type 1 Interferon Decoy Receptor Cures a Viral Disease by Restoring Interferon Signaling in the Liver

**DOI:** 10.1371/journal.ppat.1002475

**Published:** 2012-01-05

**Authors:** Ren-Huan Xu, Daniel Rubio, Felicia Roscoe, Tracy E. Krouse, Mary Ellen Truckenmiller, Christopher C. Norbury, Paul N. Hudson, Inger K. Damon, Antonio Alcamí, Luis J. Sigal

**Affiliations:** 1 Immune Cell Development and Host Defense Program, Fox Chase Cancer Center, Philadelphia, Pennsylvania, United States of America; 2 Centro de Biología Molecular Severo Ochoa, Consejo Superior de Investigaciones Científicas and Universidad Autónoma de Madrid, Madrid, Spain; 3 Department of Microbiology and Immunology, College of Medicine, Pennsylvania State University, Hershey, Pennsylvania, United States of America; 4 Poxvirus and Rabies Branch, Division of High Consequence Pathogens and Pathology, NCEZID, Centers for Disease Control and Prevention, Atlanta, Georgia, United States of America; Washington University School of Medicine, United States of America

## Abstract

Type 1 interferons (T1-IFNs) play a major role in antiviral defense, but when or how they protect during infections that spread through the lympho-hematogenous route is not known. Orthopoxviruses, including those that produce smallpox and mousepox, spread lympho-hematogenously. They also encode a decoy receptor for T1-IFN, the T1-IFN binding protein (T1-IFNbp), which is essential for virulence. We demonstrate that during mousepox, T1-IFNs protect the liver locally rather than systemically, and that the T1-IFNbp attaches to uninfected cells surrounding infected foci in the liver and the spleen to impair their ability to receive T1-IFN signaling, thus facilitating virus spread. Remarkably, this process can be reversed and mousepox cured late in infection by treating with antibodies that block the biological function of the T1-IFNbp. Thus, our findings provide insights on how T1-IFNs function and are evaded during a viral infection *in vivo*, and unveil a novel mechanism for antibody-mediated antiviral therapy.

## Author Summary

Type 1 interferons are molecules important in the defense against viruses. Orthopoxviruses encode a Type 1 interferon binding protein that acts as a decoy for the Type 1 interferon receptor. Here we show that during infection with the Orthopoxvirus ectromelia virus, the agent of mousepox, Type 1 interferons protect the liver locally rather than systemically. We also show that the Type 1 interferon binding protein of ectromelia virus attaches to uninfected cells surrounding infected foci in the liver to impair their ability to receive Type 1 interferon signaling and facilitate virus spread and disease progression. We also show that this process can be reversed and mousepox cured late in infection by treating mice with antibodies that block the biological function of the Type 1 interferon binding protein. Because the Type 1 interferon binding proteins of different Orthopoxviruses are very well conserved, the antibodies also block the biological function of the Type 1 interferon binding proteins from variola virus (the virus of smallpox) and monkeypoxvirus. Thus, our findings provide insights on how Type 1 interferons function and are evaded during a viral infection *in vivo*, and unveil a novel mechanism for antibody-mediated antiviral therapy.

## Introduction

Type 1 interferons (T1-IFNs) are cytokines produced during viral infections by most infected cells and by some uninfected cells that recognize exogenous pathogen associated molecular patterns (PAMPS) through pattern-recognition receptors such as toll-like receptors. An important function of T1-IFNs is to stimulate the transcription of interferon stimulated genes (ISGs) through the nuclear translocation of the phosphorylated Stat1 transcription factor in cells which results in increased resistance to viral infection at the cellular and organismal level [1]. Many experimental approaches use systemic infection with viruses, which results in the rapid induction of systemic T1-IFNs and, consequently, ISGs. However, this type of infection rarely occurs in nature.

To cause systemic disease, many viruses of importance to human and animal health such as viruses in the genera Orthopoxvirus (OPV, variola (VARV), monkeypox (MPXV)), Enterovirus (polio, coxsackie), Aphtovirus (foot-and-mouth disease), Rubivirus (rubella), Flavivirus (Yellow Fever, Dengue, West Nile), Rubulavirus (mumps), Morbillivirus (measles), Varicelovirus (chickenpox), and others, penetrate their hosts through disruptions of epithelial surfaces and disseminate stepwise to distant organs through a lympho-hematogenic (LH) route [2], [3]. In these cases, we do not know whether PAMPS or T1-IFNs produced at the initial sites of a viral infection can respectively stimulate T1-IFN or ISGs systemically to protect organs before the arrival of the virus or whether the induction and effects of T1-IFNs require local viral replication in the target organ.

To counteract the anti-viral effects of T1-IFNs, OPVs including ectromelia virus (ECTV, the causative agent of mousepox), variola virus (VARV, the agent of smallpox), monkeypox virus (MPXV, the agent of monkeypox) and vaccinia virus (VACV, the virus in the smallpox vaccine) encode a highly conserved T1-IFN binding protein (T1-IFN bp), an early protein that functions as a decoy to divert T1-IFN from the cellular receptor [4]–[9]. Despite that the ECTV T1-IFNbp blocks mouse IFN-α but not IFN-β [5], it is essential for its virulence [10]. Still, how and where the T1-IFNbp exerts its effects *in vivo* is not known.

It is generally assumed that the major mechanism whereby antibodies protect from viral diseases in general and OPVs in particular is through viral particle neutralization. Alternatively, Ab protection may results from Ab effector functions such as the induction of antibody dependent cellular cytoxicity (ADCC), the promotion of phagocytosis and the activation of the complement cascade to eliminate virions and/or infected cells [11]–[13]. It is well established that Abs that block secreted bacterial virulence factors such as the toxins produce by Clostridia are protective [14]. Some viral immune evasion molecules, including the T1-IFNbp of OPVs, are secreted and theoretically similarly susceptible to the action of Abs [15]. Whether Abs that block the function of these virulence factors can protect or cure viral diseases is not known. If they do, they could provide new opportunities for anti-viral intervention. We have recently shown that ECTV T1-IFNbp induces antibody (Ab) responses during infection and that, despite being an non-structural protein, immunization with recombinant T1-IFNbp protects mice from mousepox [10]. However, the mechanism of this protection remains undefined.

The pathogenesis of ECTV serves as the classic textbook example of stepwise pathogenesis [3], [16]. ECTV infects through microabrasions in the footpad, spreads *via* draining lymph nodes (D-LN) and the blood to infect the liver and spleen, and causes death 8–11 days post infection (dpi) due to acute liver failure [17]. Here we used ECTV as a model to show that local as opposed to distant infection mediates T1-IFNs production and ISG induction during infection with a virus that disseminate following the common LH route. Moreover, we demonstrate that the T1-IFNbp exerts its effects by attaching to uninfected cells p to block T1-IFN signaling. Finally, we show that Abs that block the biological activity of the T1-IFNbp cure mousepox late in infection demonstrating for the first time that Abs to a secreted immune evasion protein can cure a viral disease.

## Results

### Type 1 IFN production and signaling depends on local virus replication and is blocked in situ by the T1-IFNbp

**Figure 1:**
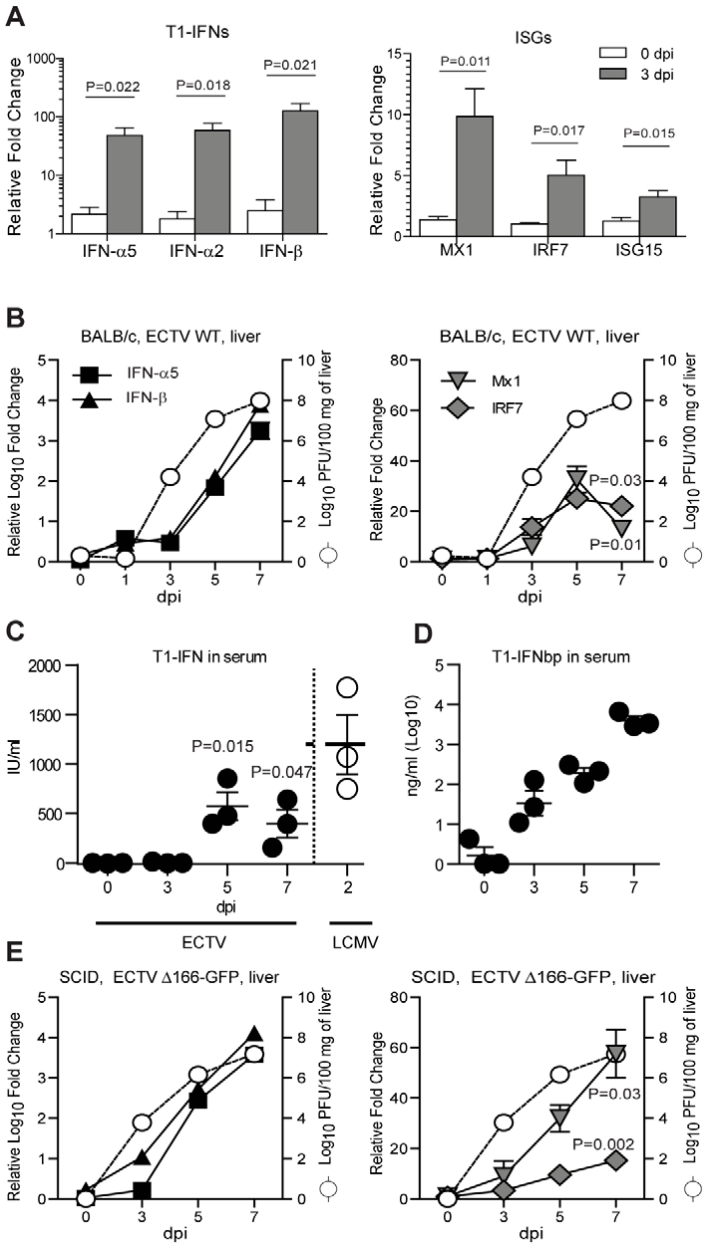
Type I IFN production and signaling depends on local virus replication and is blocked in situ by the T1-IFNbp. **A**) BALB/c mice were infected with 100 PFU ECTV WT in the footpad. At the indicated dpi, IFNs and Mx1 transcripts were determined by RT-qPCR in the D-LN. **B**) As in A, but the indicated transcripts were determined in the liver by RT-qPCR (left y axes) and virus titers were determined by plaque assay (open circles, right y axis, virus titers in upper and lower panels are identical data). **C**) BALB/c mice were infected with 100 PFU ECTV and at the indicated dpi T1-IFN in serum was determined using ISRE-Luc reporter cells. Mice infected intraperitoneally with LCMV were used as a positive control (2 dpi). **D**) BALB/c mice were infected with 100 PFU ECTV and at the indicated dpi T1-IFNbp in the sera was quantified using a sandwich ELISA and recombinant T1-IFNbp as standard. **E**) As in B, but SCID mice were infected with ECTV Δ166-GFP. All graphs show the mean ± SEM and are representative of two or three similar experiments with 3–5 mice/group in each experiment.

To determine when T1-IFN and ISG are induced during ECTV stepwise dissemination, we determined T1-IFN (IFN-β and IFN-α5) and ISG (Mx1, IRF-7 and sometimes ISG15) transcripts in organs of ECTV infected or uninfected BALB/c mice by quantitative PCR (qPCR). Preliminary experiments indicated these T1-IFNs and ISGs are representative of several other T-1IFNs and ISGs. We focused on the popliteal D-LN because it is an obligatory D-LN for ECTV spread, and on the liver, because it is the major target organ of ECTV and liver necrosis is thought to be the cause of death during acute mousepox. At 3 dpi with ECTV, transcripts for T1-IFNs and ISGs increased in the D-LN as compared to uninfected (0 dpi) mice (**Figure 1A**) and virus titers were 6.142±0.1 Log_10_ PFU/organ. At this early time point, T1-IFN and ISG transcripts had not been induced in the liver **(** **Figure 1B** **)**. Furthermore, T1-IFN was not detected in the serum using a sensitive biological assay **(** **Figure 1C** **).** This indicated that virus replication and T1-IFN production in the D-LN did not result in systemically available T1-IFN or in T1-IFN production or IFN signaling in the liver. The appearance of ISGs (**Figure 1B, right panel**) in the liver followed the appearance of virus and T1-IFN transcripts in the organ (**Figure 1B, left panel**) indicating that local virus replication and T1-IFN production are respectively required for T1-IFN and ISG induction in the liver. Similar to T1-IFN transcripts, ISG transcripts increased in the liver from 3 to 5 dpi. However, while virus loads and T1-IFN transcripts continued to increase from 5 to 7 dpi, ISG transcripts decreased (**Figure 1B**) suggesting a blockade of T1-IFN signaling in the liver of infected mice. In addition, significant T1-IFN activity was observed in the serum at 5 and 7 dpi (**Figure 1C**). This was IFN-β because the biological assay was inhibited by pre-treating the serum with anti-IFN-β but not with anti-IFN-α Ab (not shown). The absence of IFN-α activity could be due to the action of the T1-IFNbp.

We used a sandwich ELISA to test whether at different dpi, T1-IFNbp was released systemically (**Figure 1C**). At 3 dpi, the amount of T1-IFNbp in sera was variable (55±37 ng/ml) but significantly different from uninfected mice (P = 0.0251) indicating that the soluble protein percolated systemically. The amount of T1-IFNbp did not increase significantly between 3 and 5 dpi (209±57 ng/ml). However, there was a highly significant 20 fold increase (P = 0.0001) from 5 to 7 dpi (4,291±1,172 ng/ml).

To directly determine whether the T1-IFNbp was responsible for the decrease in ISG transcription in the liver at 7 dpi, we used ECTV Δ166-GFP (a mutant where EVM166, the gene coding for the T1-IFNbp, was replaced by green fluorescence protein) which is highly attenuated in BALB/c mice [10] but is lethal to severe combined immunodeficient (SCID [18]) mice. As in BALB/c mice infected with WT ECTV, the appearance of T1-IFNs (**Figure 1E, left panel**) and ISG (**Figure 1E, right panel**) transcripts in the liver of SCID mice also followed the appearance of virus. Thus, the relatively low levels of T1-IFNbp in the blood during WT ECTV infection do not appear to be responsible for the lack of systemic induction of ISG in the liver. Most likely, this is because unlike other frequently used models such as intraperitoneal infection with lymphocytic choriomeningitis virus (LCMV, **Figure 1C**, clear circles) there is no systemic T1-IFN activity during early ECTV infection. Still, different to WT ECTV infection of BALB/c mice, the transcription of ISG in the liver of SCID mice infected with ECTV Δ166 increased significantly between 5 and 7 dpi (**Figure 1E, right panel** **)**. Consequently, even though systemic T1-IFN activity was present at 7 dpi, the T1-IFNbp was able to dampen T1-IFN signaling in the liver of BALB/c mice infected with WT ECTV.

### T1-IFNbp binds to infected and uninfected cells in the liver and spleen

**Figure 2:**
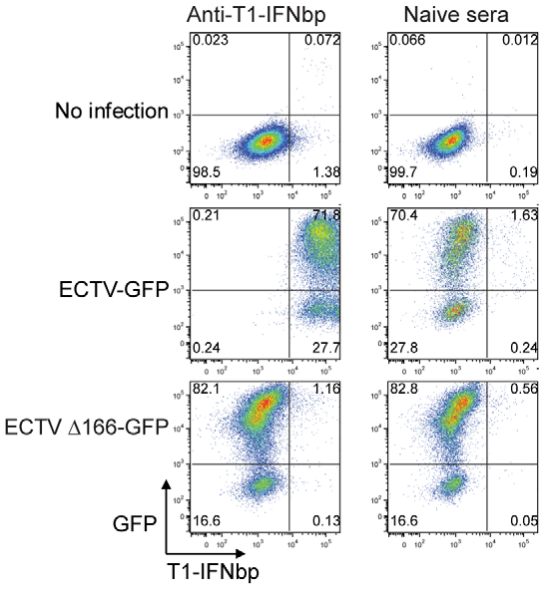
Secreted T1-IFNbp bind back to the cell surface of infected and uninfected cells *in vitro*. L929 cells were left uninfected (top panels), infected with ECTV-GFP (middle panels) or ECTV Δ166-GFP (lower panels). 6 h after infection, the presence of T1-IFNbp at the cell surface was detected using anti-T1IFNbp (left panels) or naïve control sera (right panel). The data are representative of two similar experiments.

**Figure 3:**
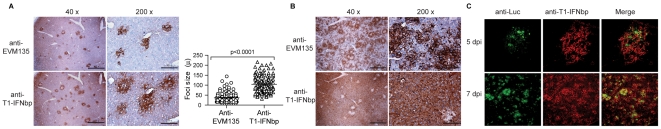
T1-IFNbp binds to infected and uninfected cells in the liver. **A**) BALB/c mice were infected with WT ECTV. At 5 dpi livers were harvested and serial sections were stained with anti-T1-IFNbp or anti-EVM135 as indicated. Data are representative of three mice/group and of two independent experiments. The dot plot represents the size of all the foci found in five randomly selected microscopy fields as detected with the two antisera at 5 dpi. **B**) As in A, but showing serial sections at 7 dpi. Data are representative of three mice/group and of two independent experiments. **C**) As in A and B, but mice were infected with ECTV-Luc and livers sections stained with anti-Luc Ab to identify infected cells (green) and anti-T1-IFNbp (Red). Data are representative of 3 mice and two independent experiments. Original magnification was 200X.

*In vitro*, the early T1-IFNbp is secreted from infected cells, but can also bind back to the surface of infected and uninfected cells [8], [19] by attaching to glycosaminoglycans at the cell membrane [20]. Consistent with these reports, when we infected L cells with 1 MOI ECTV-GFP (a recombinant ECTV expressing non-structural cytosolic GFP under the early/late 7.5 VACV promoter [21] and as virulent as WT ECTV, [10]), both, the GFP^+^ and GFP^-^ cell populations were stained by the anti-T1-IFNbp sera. Presumably, the GFP^-^ cells were not actively synthesizing viral proteins and most likely acquired T1-IFNbp from the GFP^+^ cells. As a control, L cells infected with ECTV Δ166-GFP did not stain with anti- T1-IFNbp (**Figure 2**). We tested whether a similar phenomenon occurred *in vivo*. At 3 dpi, very few cells stained with rabbit antisera toT1-IFNbp or to EVM135, the ECTV ortholog of the early/late VACV structural protein A33R [9], [22] which exclusively stains infected cells. On the other hand, at 5 dpi, both antisera stained numerous foci of cells in serial sections of the livers of BALB/c mice. While coincident in space, the foci stained with anti-T1-IFNbp were significantly larger than those stained with anti-EVM135 suggesting that the T1-IFNbp spread further than the virus itself (**Figure 3A** **)**. Consistent with the large quantities of T1-IFNbp in the serum, at 7 dpi, the liver appeared saturated when stained with anti-T1-IFNbp even though some areas did not stain with anti-EVM135 (**Figure 3B**). We also infected mice with ECTV expressing cytosolic firefly luciferase (ECTV-Luc) controlled by the 7.5 promoter as a surrogate of viral protein. This allowed us to perform two-color immunoflourescence using rabbit anti-T1-IFNbp and goat anti-Luc to reveal T1-IFNbp bound to uninfected cells surrounding liver infected foci at 5 and 7 dpi (**Figure 3C**). A similar phenomenon was also observed in the spleen, which is also a target of ECTV (**Figure S1**). Thus, as in tissue culture, secreted T1-IFNbp binds to the surface of uninfected cells *in vivo.*

### T1-IFNbp Abs bind to cells in and surrounding infected foci in vivo and cure mice from lethal mousepox

**Figure 4:**
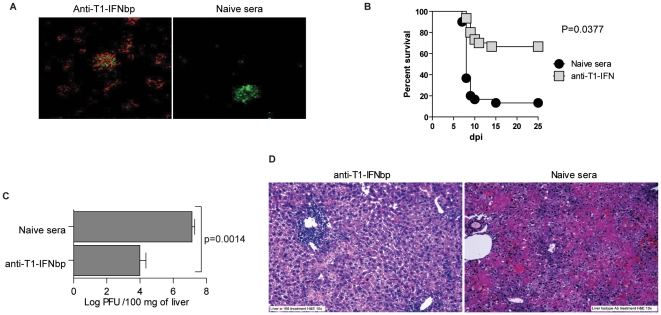
T1-IFNbp Abs bind to cells in and surrounding infected foci in vivo and cure mice from lethal mousepox. **A**) Mice were infected with 100 PFU ECTV-Luc. At 5 dpi they were treated with 200 µl (∼30 mg/ml protein) anti-T1-IFNbp or naïve sera. 16 h later the livers were harvested and stained with anti-rabbit IgG (red) and anti-luc (green). **B**) BALB/c mice were infected with 100 PFU of WT ECTV in the footpad. At 5 dpi, mice were treated i.p. with 200 µl rabbit antisera to the T1-IFNbp or, as a control, with naïve sera on the day of infection and monitored for survival. Data are representative of three similar experiments with groups of 10 mice. P values are vs. naïve sera. **C**) BALB/c mice were infected with WT ECTV. At 5 dpi they were treated with 200 µl anti-T1-IFNbp rabbit sera. Livers were harvested 2 dpt and virus loads determined by plaque assay. Graph shows the mean +/− SEM for 5 individual mice/group and two independent experiments. **D**) As in C but livers sections stained with H&E. Data are representative of five mice/group and two independent experiments.

It was of interest to test whether T1-IFNbp Ab could also bind to the surface of cells in and surrounding infected foci in the liver following *in vivo* administration. For this purpose, BALB/c mice infected with ECTV-Luc were given 200 µl anti-T1-IFNbp or control naive sera at 5 dpi and their livers stained with anti-rabbit and anti-luc 16 h later. We found that the cells within and surrounding infected foci in the liver were decorated with rabbit IgG in mice treated with T1-IFNbp antisera but not with control sera (**Figure 4A**).

Given that T1-IFNbp Ab bound to infected foci *in vivo*, we tested whether T1-IFNbp antisera could prevent or cure mousepox. We inoculated BALB/c mice with rabbit T1-IFNbp antisera on different days post infection (dpi). The antisera significantly protected from lethality when given as late as at 5 dpi (**Figure 4B**). Treatment with T1-IFNbp antisera at 5 dpi significantly reduced virus titers in the liver (**Figure 4C**) and liver necrosis seen as dark pink areas devoid of nuclei (**Figure 4D**) at 2 days post treatment (dpt).

### Identification of a mAb that inhibits the biological activity of ECTV T1-IFNbp

**Figure 5:**
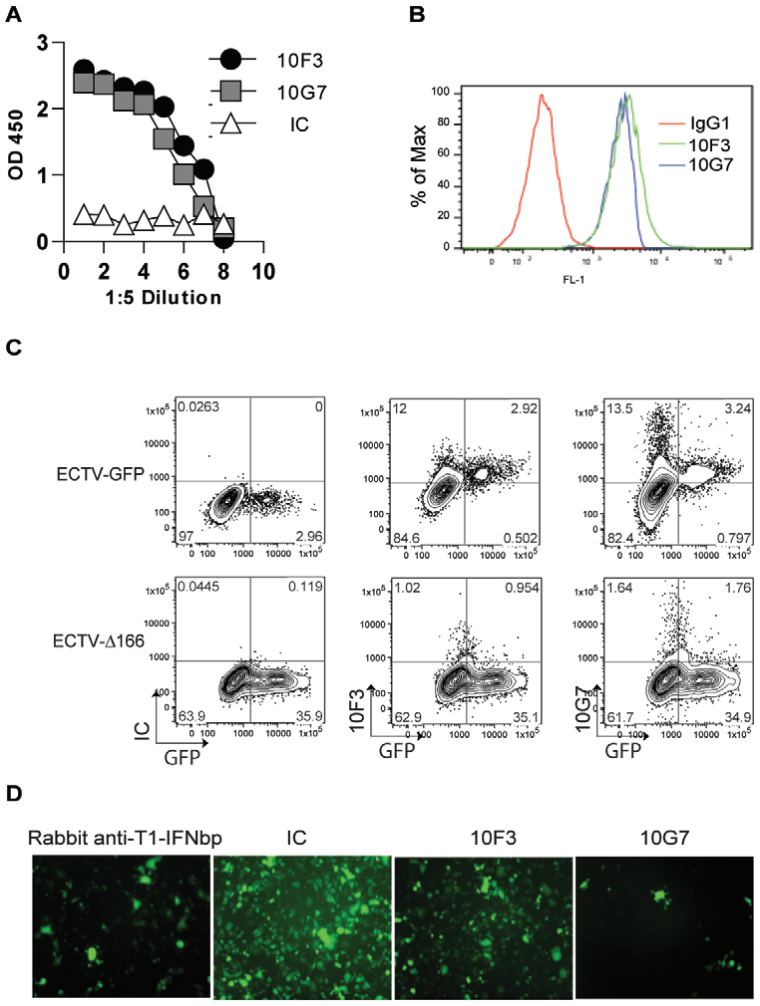
Identification of a mAb that inhibits the biological activity of ECTV T1-IFNbp. **A**) ELISA plates were covered with recombinant T1-IFNbp and the presence of the protein was detected with the indicated mAbs or isotype control (IC). Data are representatives of 2 independent experiments with similar results. **B**) L929 cells (10^5^) were incubated with 10 ng recombinant T1-IFNbp at 37°C for 1 h, thoroughly washed, and the binding of mAb 10G7 (blue line) or 10F3 (green) to the cell surface was analyzed by flow cytometry. **C**) L929 cells were infected with ECTV-GFP (upper panels) or ECTV-Δ166-GFP (lower panels) at MOI of 0.05 for 16 h. GFP expression and the binding of IC, 10G7 or 10F3 mAbs were determined by flow cytometry as indicated. **D**) 10 ng of recombinant ECTV T1-IFNbp was incubated with 10 µl rabbit anti-IFNbp or 10 ng of indicated mAbs for 30 min at 37°C, incubated with 1U mIFN-α for another 30 min at 37°C. The cocktail was then added to L929 cells in 96-well plates. The cells were incubated with this cocktail for 24 h, infected with VSV-eGFP at MOI of 0.1 for 16 h and observed for GFP fluorescence under the microscope.

The protection observed with the T1-IFNbp antisera could be due to an ability to restore T1-IFN signaling (i.e. by inhibiting the biological activity of the T1-IFNbp) and/or by traditional Ab effector mechanisms such as antibody dependent cellular cytotoxicity (ADCC), phagocytosis, or complement activation [11]–[13]. We identified two mAbs, 10F3 and 10G7 that bound to recombinant ECTV T1-IFNbp with similar efficiency in ELISA (**Figure 5A**) and at the surface of cells incubated with recombinant ECTV T1-IFNbp (**Figure 5B**) or infected with ECTV-GFP (**Figure 5C**). However, 10G7 fully blocked the ability of ECTV T1-IFNbp to inhibit the antiviral function of mouse IFN-α as determined in vesicular stomatitis virus (VSV) inhibition assays while 10F3 had a very moderate inhibitory effect (**Figure 5D**). Both mAbs were IgG1, an isotype known to have poor effector function in the mouse [23], [24].

### The T1-IFNbp inhibitory mAb 10G7 restores T1-IFN signaling in vivo and cures mousepox

**Figure 6:**
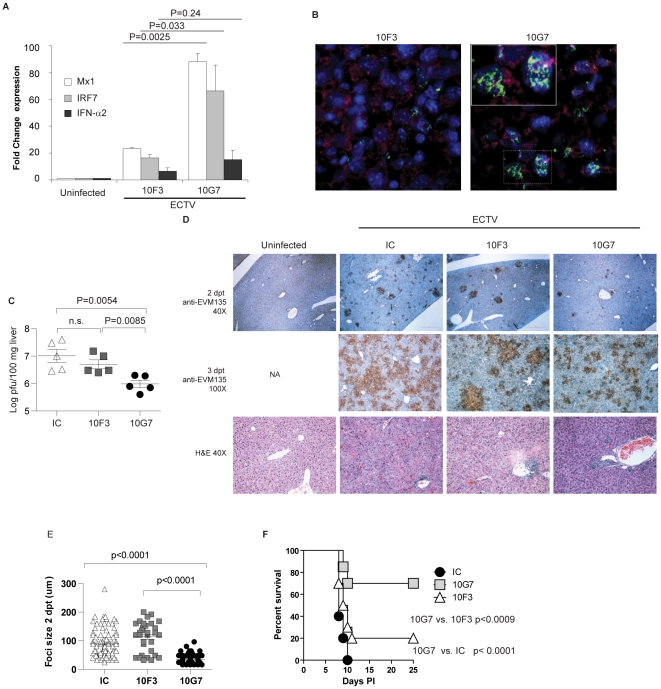
The T1-IFNbp inhibitory mAb 10G7 restores T1-IFN signaling in vivo and cures mousepox. **A**) BALB/c mice were infected with 100 PFU of ECTV in the footpad. At 5 dpi, mice were treated with the indicated mAbs i.p. One day later (a time when virus loads remain the same in all mice), the indicated transcripts in the livers were determined by RT-qPCR. Data are representative of two similar experiments with 3–5 mice/group. **B**) BALB/c mice were infected with ECTV-Luc in the footpad. At 5 and 6 dpi, mice were treated with the indicated mAbs. 2 dpt the livers were harvested and sections were stained with anti-Phospho-Stat1 (green), anti-Luciferase (red) and with DAPI to reveal nuclei. For the picture on the right (10G7 treated), the small rectangular area at the bottom is shown at higher magnification in the upper-left rectangular area. Data are representative of two similar experiments. **C**) As in A, but livers were harvested 2 dpt and viral load determined by plaque assay. Graph shows individual mice with the mean ± SEM and is representative of three similar experiments. **D**) As in A, but livers were harvested 2 or 3 dpt as indicated. Immunohistochemistry with anti-EVM135 sera (top and middle panels) and H&E staining (lower panels). **E**) Size of individual foci and the mean ± SEM 2 dpt in five randomly selected microscopy fields from D. **F**) BALB/c mice were infected with 100 PFU of WT ECTV in the footpad. At 5 dpi, mice were treated with indicated mAb i.p. and survival was monitored. Data are representative of 3 independent experiments each with 10 mice/group.

**Figure 7:**
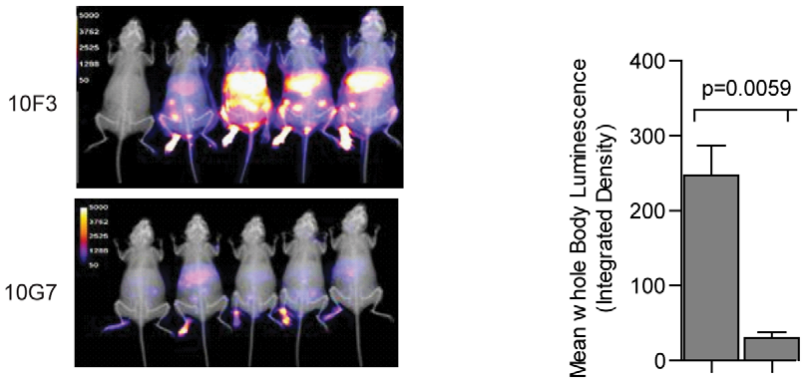
Treatment with blocking 10G7 but not with non-blocking 10F3 decreases overall virus loads as determined by whole body imaging. BALB/c mice were infected with 300 pfu of ECTV-Luc in the footpad, at 5 dpi, the mice were treated with indicated mAbs IP and imaged 2 dpt for light emission using a Carestream *In vivo* instrument for bioluminescence detection (left) The mouse on the top-left is an uninfected control. The bar graph (right) shows the mean ± SEM quantitative luminescence intensity of the two groups. The experiment is representative of two similar experiments.

To determine whether 10G7 and 10F3 also differed in their ability to inhibit the biological function of T1-IFNbp *in vivo*, BALB/c mice were infected with ECTV and at 5 dpi treated with inhibitory 10G7 or poorly-inhibitory 10F3. At one dpt, the virus titers (not shown) and T1-IFN transcripts in the livers of 10G7- and 10F3-treated mice were similar. However, ISG transcription in mice treated with inhibitory 10G7 was significantly increased as compared to mice treated with 10F3 (**Figure 6A**). In other experiments, mice treated with isotype control also failed to upregulate T1-IFN and ISG transcripts (not shown) The upregulation of ISGs after 10G7 treatment was not due to direct stimulation of IFNAR by 10G7 because uninfected mice treated with 10G7 did not upregulate Mx1 (**Figure S2**). Moreover, phosphorylated Stat1 in the nuclei of infected hepatocytes was readily apparent in the livers of mice treated with 10G7 but not with 10F3 (**Figure 6B**). At 2 dpt, mice treated with inhibitory 10G7 had significantly lower virus titers in their livers as compared with mice treated with inhibitory 10F3 or isotype control in plaque assays (**Figure 6C**) suggesting that the increase in ISG transcription resulted in improved antiviral state. Moreover, immunohistochemistry with anti-EVM135 sera at 2 and 3 dpt (**Figure 6D** **upper and middle panels and** **Figure 6E**)) showed that the foci in mice treated with inhibitory 10G7 were smaller than those of mice treated with 10F3 or IC suggesting reduced virus spread. Also, at 3 dpt the livers of mice treated with inhibitory 10F3 or IC were necrotic but not those from mice treated with inhibitory 10G7 (**Figure 6D** **, lower panels**). Finally, the survival of mice treated with inhibitory 10G7 was significantly higher than of those treated with 10F3 or IC (**Figure 6F**). Thus, while 10F3 and 10G7 T1-IFNbp mAbs bound equally well to cell surfaces and similar numbers of inflammatory cells were recruited to the livers of treated mice, only the inhibitory mAb 10G7 rescued T1-IFN signaling, decreased liver damage and virus loads, and prevented lethal mousepox. Decreased virus loads after 10G7 treatment was also observed when mice were infected with ECTV-Luc and the virus loads visualized by whole animal imaging (**Figure 7**). Additionally, at 2 dpt significantly more leukocytes and more CD8^+^ T cells were recovered from the livers of mice treated with inhibitory 10G7 and 10F3 as compared with uninfected mice. On the other hand, no significant differences were observed between groups of mice treated with 10G7, 10F3 or IC. Also, no significant differences were observed in other immune populations (**Figure S3**). Thus, mAb treatment did not affect the infiltration of leukocytes in the liver that normally occurs during ECTV infection.

### The ECTV T1-IFNbp inhibitory mAb 10G7 inhibits the biological activity of the T1-IFNbp from OPVs important for human health

**Figure 8:**
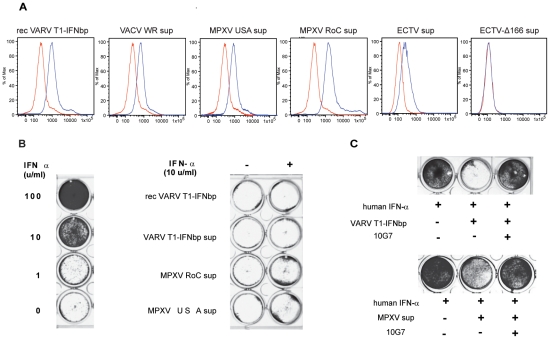
The ECTV T1-IFNbp inhibitory mAb 10G7 inhibits the biological activity of the T1-IFNbp from OPVs important for human health. A) Hela cells were incubated with 10 ng recombinant VARV T1-IFNbp,UV treated supernatants from cells infected with VACV WR, or gamma-irradiated supernatants of cells infected with MPXV USA or MPXV Republic of Congo (RoC) strains as indicated. After 30 min the cells were thoroughly washed, incubated with mAb 10G7 (blue line) or IC (red line) for 1 h followed by FITC-anti-mouse IgG and flow cytometry analysis. All data are representative of 2 or 3 independent experiments with similar results. B) Tissue culture media (TCM, RPMI 10% fetal calf serum), 10 ng of recombinant VARV T1-IFNbp in TCM, 100 µl of supernatant of insect cells expressing recombinant VARV T1-IFNbp in TCM, or the indicated irradiated TCM supernatant from cells that had been infected with the indicated viruses were pre-incubated with the indicated amounts of hIFN-α for 1 h. The cocktails were then added to Hela cells in 24 well plates. Following 24 h incubation at 37°C, the cells were infected with VSV at a MOI of 0.1 for 24 h, fixed, and stained with crystal violet. Left and right panels correspond to the same plate but were separated to facilitate labeling of the figure. C) 10 ng of recombinant VARV T1-IFNbp or 100 µl of irradiated supernatant from cells that had been infected with MPXV USA were incubated with 10 ng mAb 10G7. After 30 minutes, 10 U/ml hIFN-α were added to the mixture and incubated for 1 h at 37°C. The cocktail was then added to Hela cells in 24 well plates. Following 24 h incubation at 37°C, the cells were infected with VSV at a MOI of 0.1 for 24 h, fixed, and stained with crystal violet. All data are representative of 2 or 3 independent experiments with similar results.

Given that 10G7 can inhibit the biological activity of T1-IFNbp from ECTV and protect from disease, it was of interest to determine whether 10G7 can also block the biological activity of T1-IFNbp from OPVs important to human health. We found that 10G7 recognized cells pre-incubated with recombinant T1-IFNbp from the OPV variola virus (VARV, the agent of human smallpox), or supernatants of cells infected with the OPVs vaccinia virus (VACV, the virus in the smallpox vaccine) or monkeypox virus (MPXV, endemic in human populations of central Africa) (**Figure 8A**). Furthermore, while the antiviral activity of 10 U/ml Human IFN-α (hIFN-α) was inhibited by recombinant VARV T1-IFNbp or supernatants of MPXV infected cells ([4], [6], [19], [25] **Figure 8B**); pre-incubation of VARV T1-IFNbp or MPXV supernatants with mAb 10G7 blocked their ability to inhibit the antiviral function of hIFN-α (**Figure 8C**) demonstrating the ability of 10G7 to block the biological function of T1-IFNbp from different OPVs.

## Discussion

The results presented here provide novel information regardingT1-IFN induction and protection *in vivo*. While the induction of T1-IFNs *in vivo* following systemic (intravenous (i.v.) or intraperitoneal (i.p.) administration of viruses has been studied to a great extent [26]–[28] and their ability to induce an antiviral response is well known [1], we still lack an understanding of how T1-IFNs and ISGs are temporally induced and protect from disease during the course of the many viral infections that follow a stepwise mode of LH dissemination [16]. The induction of T1-IFN genes depends on cells sensing viral infection. Cells recognize Pathogen Associated Molecular Patterns (PAMPs) of viruses (in most cases nucleic acids) by means of Pathogen Recognition Receptors (PRR) expressed at the plasma membrane (e.g. Toll Like Receptor (TLR)2, TLR4), in endosomes (TLR3, TLR7, TLR9) or in the cytosol (RIG-I, MDA5, DAI). [29]–[31]. Signaling through PRRs culminate in the activation of specific members of the IRF family of transcription factors, most notably IRF3, IRF7, NF-κB and c-jun which stimulate the T1-IFN promoters. During stepwise infection, T1-IFNs could act on vital target organ indirectly. For example, they could induce ISGs and help orchestrate the innate and adaptive immune response in the D-LN thereby curbing virus spread to the target organ. As we have previously shown, this is a major mechanism whereby NK [32]–[34] and memory CD8+ T cells [35] protect mice from mousepox. Alternatively, T1-IFNs could directly induce ISGs in the target organ and/or contribute to the recruitment of immune cells. In this case, the protection of the target organ could result from the T1-IFN produced at the primary site of infection that is distributed systemically, or from the T1-IFN produced locally in the target organ from PAMPS either distributed systemically or locally produced. Here we have used the classical ECTV model of LH spread to show that during ECTV infection, T1-IFN signaling in the liver (the target organ) strongly correlates with resistance to disease. We also show that ISG induction in the liver correlates with T1-IFN transcription in the liver but not in the D-LN suggesting that T1-IFN signaling in the liver is reliant on local T1-IFN production. Moreover, we demonstrate that the induction of T1-IFN in the liver depends exclusively on local viral replication. This suggests that PAMPS produced in the footpad or in the D-LN do not distribute systemically to the liver before virus arrival. It should be noted, however, that poxviruses excel in the number of immune evasion proteins that affect innate immunity [36]. Hence, it is possible that during other viral infections where T1-IFN production and signaling is not targeted by the virus, the systemic distribution of T1-IFN may have a more important role in distant ISG induction.

Our work also impinges on our understanding of viral immune evasion. During the past few years there has been much progress towards the characterization of virally encoded immune evasion genes. While the cellular and molecular mechanisms whereby many of these evasion molecules operate are well known [36]–[39], we still have an incomplete understanding on how they subvert the immune response *in vivo*. We have previously shown that the OPV T1-IFNbp is secreted from infected cells and binds back to cell surfaces [19] by attaching to glycosaminoglycans at the cell membrane [20]. Whether this also occurs *in vivo* and is significant for viral virulence remained unknown. Our experiments reveal that the T1-IFNbp produces its evasive effect at least in part by attaching to uninfected liver cells surrounding infected foci, thereby precluding their ability to signal through the T1-IFN receptor. Of interest, the ECTV T1-IFNbp does not block IFN-β although ECTV infection induced IFN-β transcription. A remaining question is why IFN-β was insufficient to induce high levels of ISGs; our data may indicate that *in vivo*, IFN-α and IFN-β have different functions.

Our results also have implications to our understanding of Ab-mediated protection from viral disease. The most commonly accepted mechanism of Ab protection is viral particle neutralization [11]–[13]. Indeed, it has been suggested that this is the mechanism whereby the smallpox vaccine protects [40]. However, while clinical data showed that protection from smallpox correlated with Ab neutralization, the same investigators could not find a causal association between neutralizing Ab titers and protection against smallpox [41], [42]. These findings suggests that mechanisms other than viral particle neutralization may be involved in protection by the smallpox vaccine. In support of this, Benhnia et al. recently demonstrated that mice can be protected from VACV by prior administration of Abs to the structural protein B5R and that this protection relied in complement activation [13], [43], a well known Ab effector mechanism. Here we show that Abs can protect from and cure advanced systemic OPV disease by a previously unsuspected mechanism: inhibiting the function of an immune evasion protein. Of interest, while it is known that polyclonal antibodies in the form of convalescent sera and vaccinia immunoglobulin (VIG) are protective pre- and soon after exposure to VARV, the possible mechanisms of this protection remain unknown [12], [13]. We show that Abs to the T1-IFNbp cure mousepox even when administered as late as at 5 dpi suggesting that Abs to the T1-IFNbp may play a role in protection not only by the smallpox vaccine but also by VIG because, at least in mice, anti T1-IFNbp are present in sera following VACV vaccination [10]. Thus, our experiments uncovered a novel mechanism of Ab mediated protection. We have previously shown the importance of the T-1 IFNbp in ECTV pathogenesis [10]. Whether Abs to other secreted immunoregulatory viral protein could have a similar effect will likely depend on whether they play an essential role in pathogenesis or not and remains to be studied.

It is interesting to note that while resistance to primary infection with ECTV requires T1-IFN function, resistance to secondary infection does not as IFNAR1 deficient mice immunized with attenuated ECTV or VACV resisted a challenge with WT ECTV [44], [45]. This suggests that the main role of T1-IFNs in protection is to control the virus until an adaptive response is generated and thereafter become irrelevant. Hence, it is likely that Abs to the T1-IFNbp also have a similar effect.

Drugs being tested for the treatment of OPV infections are ST-246 [46], which targets VACV F13L protein and its orthologs in other OPVs to inhibit the egress of extracellular virions from cells, and CMX001, an oral ether-lipid analogue of the acyclic nucleoside phosphonate Cidofovir [47], that target the viral DNA polymerase. These two drug types have been very effective for the treatment of various OPVs in several animal models [48]–[54]. CMX001 has been shown to cure intranasal ECTV infection when treatment was started as late as at 5 dpi [51]. Still, there is the caveat that OPVs could naturally develop resistance, or that resistant viruses could be artificially created. Indeed, VACV resistant to Cidofovir and its derivatives has been demonstrated [55]–[59]. Similarly, cowpox virus resistant to ST-246 has been isolated [46]. Thus, more than one or two anti-poxvirus drugs directed towards different targets are needed.10G7 mAb or similar T1-IFNbp mAbs could be exploited to treat humans against OPV infections because OPVs that affect humans encode a T1-IFNbp and at least three of them (VACV,VARV and MPXV) are inhibited by 10G7. In addition, a strategy of using mAbs to inhibit secreted immune evasion proteins important for viral pathogenesis could be explored to prevent and treat infections with any other virus that encode proteins of this kind.

## Materials and Methods

### Ethics statement

This study was carried out in strict accordance with the recommendations in the Guide for the Care and Use of Laboratory Animals of the National Institutes of Health. All protocols were approved by Fox Chase Cancer Center's Institutional Animal Care and Use Committee.

### Mice

BALB/c female mice were purchased from Taconic Farms. SCID mice in a BALB/c background were bred at FCCC Laboratory Animal Facility. All mice used in experiments were 5–12 week old.

### Cells, viruses and recombinant proteins

Media and cells were as previously described [10], [60], [61]. For antibody production, hybridomas were grown in a Celline devise (BD) using protein free MAb Medium (BD) as recommended by the manufacturer. The mAbs were purified from the medium using ammonium sulfate precipitation and purity confirmed by SDS-PAGE gel.

Stocks of ECTV Moscow strain (ATCC VR-1374) were propagated in tissue culture as previously described [10]. Virus titers were performed in BS-C-1 cells. Stocks of VSV Indiana VSV-*eGFP* were a gift of Dr. S. Balachandran. They were expanded in BHK cells and virus titers were determined in Vero cells by plaque assay as previously described [10].

For the generation of recombinant ECTV expressing firefly luciferase (Luc, GenBank accession number AAL30790), we used homologous recombination as previously described using ECTV-GFP [10] as host virus to replace GFP for Luc. Non-fluorescent plaques were purified 10 times, expanded, and the insertion sequenced with primers flanking the site of homologous recombination. This virus was as pathogenic as WT virus in LD50 experiments.

Mice were infected in the footpad with 100 PFU ECTV WT or Luc as indicated. For the determination of survival, the mice were monitored daily. To avoid unnecessary suffering, mice were euthanized and counted as dead if imminent death was certain. For virus titers mice were infected with 100 PFU ECTV. Mice were euthanized when indicated and whole LNs or 100 mg of liver were homogenized in PBS using a Tissue Lyser homogenizer (Qiagen). Virus titers were determined on BS-C-1 cells in 6-well-plates as before [10], [60], [61].

MPXV RoC (2003 358) and MPXV USA (2003 044) (Center for Disease Control, USA) were incubated on a BSC-40 monolayer (7.7×10^6^ cells) at a MOI of 1 for one hour at 36 ^o^C, 6% CO2 in 1 ml of Opti-MEM. After one hour the infectious inoculum was removed, replaced with 2 ml Opti-MEM and incubated for 18 hours. The supernatants were collected and spun at 1000×g for 10 min. in a tabletop microcentrifuge to remove cell debris. After transfer to a new microcentrifuge tube, the supernatants were stored at −70 ^o^C. The samples were subjected to 4.4×10^6^ rads of gamma rays for 4 hours on dry ice and stored at −70°C until use. Production of recombinant T1-IFNbp from ECTV and VARV was exactly as described previously [6], [10].

### Preparation of polyclonal Abs and monoclonal Abs to T1-IFNbp

Rabbits were immunized three times at 1 month interval with recombinant T1-IFNbp [10] or EVM135 [62] in incomplete Freund's adjuvant, sera were obtained 1 month after last injection. Antisera were evaluated for antibodies by ELISA.

To generate mouse hybridomas producing T1-IFNbp specific mAbs, BALb/c mice were immunized three times with 50 µg recombinant T1-IFNbp s.c. in incomplete Freund adjuvant. After one month of rest, the mice were boosted with 10 µg T1-IFNbp in PBS intravenous and their spleens fused the next day using standard hybridoma procedures at the FCCC Hybridoma Facility. The initial screening of mAb against T1-IFNbp was performed by ELISA. Positive mAbs were further analyzed for their ability to block T1-IFNbp using a VSV inhibition assay.

### ELISA assays

To compare the binding of Binding of mAbs to T1-IFNbp the T1-IFNbp was determined by ELISA assay was performed with immobilized T1-IFNbp as previously [10] but using dilutions of mAbs rather than serum.

For the detection of T1-IFNbp in serum, we used a sandwich ELISA. For this purpose, high-binding 96-well plates (Corning) were coated with mouse anti-T1-IFNbp (10G7, 50ng/well) at 4°C overnight. After washing and blocking, diluted sera (1∶10 dilution in PBS) were added to each well, and incubated at 37°C for 1 hr. After washing, secondary rabbit anti-T1-IFNbp serum (1∶1000 in PBS) was added and incubated at RT for 2 hr. The plates were washed four times and incubated with HRP conjugated anti-Rabbit IgG (KPL, 100 ng/well) at RT for 1 hr. Following washing, signal was developed with 100 ul of TMB (Sigma, USA) and the reaction was stopped by adding 0.5N sulfuric acid. The OD was determined at 450 nm using a multiwell plate reader. For quantification, a serial dilution (10 ug to 1 pg/ml) of recombinant T1-IFNbp was included in the same plate.

### Detection T1-IFN in serum

Concentrations of biologically active T1-IFN in serum were measured using an ISRE- (interferon stimulated responsive element) luc reporter assay as described [63]. Briefly, 1∶10 diluted sera were overlaid on L929 ISRE-Luc reporter cells (a gift from Dr. Russell Vance, University of California, Berkeley) in a 96-well plate and incubated overnight at 37°C. L929 ISRE-Luc reporter cells were lysed and the luciferase activity was measured by adding firefly luciferin substrate (Agilent Technologies) and measuring luminescence in a 96-well plate reader. Recombinant IFN-β (PBL Interferon Source) was included in the same plate for quantification.

### VSV inhibition assays

The VSV inhibition assays were modified from those described before [10]. Briefly, 10 µl of mIFN-α (0.1 I/ul, PBL) was incubated or not with recombinant T1-IFNbp (10 ng/ml) or with T1-IFNbp and the indicated mAbs (100 µl of supernatant) for 1 h at 37°C. IFN-α or the indicated mixtures were then added to L929 cells in a 12-well-plate and incubated at 37°C for 24 h to induce (or not) an antiviral state. The cells were then infected with VSV-*GFP* (at MOI of 0.01 for 16 h) or VSV (at MOI of 0.01 for 48 h). Protection or lack of protection of the cells by the mIFN-α was assessed by determining the expression of GFP under fluorescent microscope or by staining with crystal violet.

The blockade of the VARV and MPXV T1-IFNbps by the mAbs were determined similarly but using HeLa cells in 24-well plates instead of L929 cells in 12 well plates, recombinant human IFN-α (PBL) instead of mouse IFN-α, and recombinant VARV T1-IFNbp or irradiated supernatant of BS-C-1 cells that had been infected with the indicated strains of MPXV instead of ECTV T1-IFNbp. In this case, protection of the cells was determined by staining with crystal violet.

### Flow cytometry

To detect T1-IFNbp expression at the cell surface, 10^6^ L929 cells were infected with ECTV-*GFP* or ECTV-Δ166-*GFP* at MOI of 1 for 24 h, the next day, the cells were trypsinized and incubated with 100 µl of 1/1000 T1-IFNbp antisera or naïve control serum, or incubated with 10 ng of mAbs (10F3 and 10G7) in 100 µl PBS for 1 h at 37°C. The cells were then washed, stained with PE-conjugated goat anti-rabbit IgG Ab or PE-conjugated goat anti-mouse IgG Ab for 30 min, respectively and analyzed using an LSRII flow cytometer (BD).

For detection of mAb binding to recombinant T1-IFNbp attached to the surface of uninfected cells, L929 cells were incubated with 10 ng of recombinant T1-IFNbp at 37°C for 1 h, washed, incubated with 10 ng of mAb (10 F3 or 10 G7) for 1h, washed again and stained with PE goat anti-mouse IgG Ab.

For the detection mAb binding to recombinant VARV T1-IFNbp [6] and supernatant of MPVX infected cells, HeLa cells were incubated with 10 ng of recombinant VARV T1-IFNbp or 100 ul of irradiated supernatant from BS-C-1 cells that been infected with the indicated strains of MPVX for 1 h at 37°C, washed, incubated with 10 ng of mAb (10 F3 or 10 G7) for 1 h at RT, washed again and stained with PE conjugated goat anti-mouse IgG Ab for 30 min washed and analyzed by flow cytometry.

For the analysis of splenocytes and liver infiltrating leukocytes, BALB/c mice were infected with 100 PFU of ECTV. At 5 dpi, 200 ug of indicated mAb were injected i.p. 2 dpt, mice spleens and livers were harvested. Spleens were made into single cell suspensions and infiltrating lymphocytes were isolated from the livers using a Percoll gradient as previously described [64]. Cells were surface stained with Cy7PE-anti-CD8a, APC-anti-CD49b, PE-anti-CD4 and Cascade Blue anti-CD3 (Biolegend) and intracellularly with PE-anti-IFN-γ (Biolegend) and cy5.5PE-anti human Granzyme B (Invitrogen) as described previously [10], [60], [61]. Flow cytometry was performed using an LSRII flow cytometer (BD) and Flowjo software for analysis.

### Histology and immunohistochemistry

These were performed by standard procedures. Briefly, mouse livers were fixed with formalin and embedded in paraffin. 5 µM sections were stained with rabbit anti-EVM135 or T1-IFNbp antisera diluted 1∶500, followed by biotin goat anti-rabbit and extravidin-peroxidase each for 30 min. The slides were revealed using chromogen-DAB (Sigma) as substrate and counter stain with Gill's Hematoxylin. For double immunohistochemistry staining, BALB/c mice were infected with ECTV-luc for 5 days; liver sections were prepared and stained with the above procedure in two steps. In the first step, sections were stained with goat anti-Luciferase Ab and anti-goat IgG-HRP secondary and chromogen-DAB (Sigma) as the substrate. In the second step, sections were stained with rabbit antiserum to T1-IFNbp and anti-Rabbit IgG-HRP, the substrate was VIP (Vector Lab SK-4600).

For immunofluorescent double staining of luciferase and T1-IFNbp, mice were infected with ECTV-Luc rather than WT ECTV. Livers were immersed in PBS and snapped frozen in liquid nitrogen. 10 µM cryosections were fixed in 95% acetone at −20°C for 10 min, air dried at room temperature, and sequentially stained with goat anti-luciferase (Invitrogen) (1∶400), rabbit anti-T1-IFNbp antiserum (1∶400) for 60 min at RT, anti-goat Alexa Fluor 488 and anti-Rabbit Alexa fluor 555 (Invitrogen) at 1∶500 dilution for 60 min. after several washes, slides were mounted under coverslips with one drop of Fluoromount G.

For immunofluorescent double staining of luciferase and phosphor-Stat1, mice were infected with 1000 PFU ECTV-luc and treated at 5 and 6 dpi with 200 µg of the indicated mAbs. At 7 dpi, the livers were fixed with 4% paraformaldehyde for 30 minutes, washed and permeabilized with 1% Triton X-100. Slides were stained with goat anti-luciferase and rabbit anti-phospho Stat1 overnight. After several washes cells were stained with donkey anti-goat-DyLight 488 and donkey anti-rabbit-Alexa fluor 647 (both from Jackson ImmunoResearch) for 90 minutes, and then washed before mounting in Fluromount containing DAPI.

For detection of distribution of rabbit Ab *in vivo*, BALB/c mice were infected with ECTV-*Luc* in the footpad. At 5 dpi, 200 µl of antisera were injected i.p., 16 h later, mice were sacrificed and livers were snap frozen and cut into 10 um sections. Following fixation, sections were stained with Alexa-555 conjugated anti-rabbit IgG (Invitrogen) and goat anti-luciferase for 30 min and Alexa-488 conjugated anti-goat IgG Ab for another 30 min.

### Bioluminescent imaging luciferase activities in vivo

*In vivo* bioluminescent imaging was performed using a Carestream In-Vivo Multispectral FX PRO Imaging System (Carestream healthcare). Briefly, BALB/c mice were infected with 300 PFU of ECTV-*luc* in the footpad, at 5 dpi, mice were treated with indicated Ab i.p., 2 dpt, the mice were anesthetized using ketamine (70 mg/kg of body weight) and xylazine (7 mg/kg of body weight) and 150 mg/kg of D-luciferin substrate was administered i.p. exactly 10 min before acquisition. Luminescence was captured with an exposure time of 10 s. Five mice were imaged at each time. The mean luminescent intensity was determined using Carestream's Molecular Imaging software.

### Reverse transcriptase quantitative PCR (RT-qPCR)

RNA was isolated from organs using Trizol reagent (Invitrogen) according to manufacturer's instructions. Total RNA was treated with DNase I (Qiagen) and further purified using the RNeasy Mini Kit (Qiagen). 2 µg of total RNA samples were reverse transcribed using the High Capacity cDNA Reverse Transcription Kit (Applied Biosystems). 1 ng cDNA was amplified by real time PCR using TaqMan Probes for ifna5 (ID:Mm00833976_s1), ifna2 (ID:Mm00833961_s1), ifnb1 (ID:Mm00439546_s1), Mx1 (ID:Mm00487796_m1) and IRF7 (ID:Mm00516793_g1), and GAPDH (ID:Mm99999915_g1) as an internal control for normalization. Each sample was run in 20 ul reaction using TaqMan Universal PCR Master Mix. Reactions were performed in an ABI real time PCR 7500 (Applied Biosystems, Foster City, CA). Ratios of mRNA levels to control values were calculated using the ΔCt method (2^-ΔΔCt^) at a threshold of 0.02 [65]. All data were normalized to control GAPDH. PCR conditions used: hold for 10 min at 95°C, followed by 40 cycles of 15 s at 95°C and 60 s at 60°C.

### Statistics

Statistical analyses were performed using Prism software.

## Supporting Information

Figure S1 **T1-IFNbp binds to infected and uninfected cells in the spleen. A)** BALB/c mice were infected with 100 PFU ECTV-Luc. At 5 dpi spleens were harvested and frozen sections stained with anti-Luc Ab to identify infected cells (green) and anti-T1-IFNbp (red). Data are representative of 3 mice and two independent experiments (the original magnification was 200X).
(TIF)

Figure S2 **The T1-IFNbp mAbs do not activate IFNAR signaling directly.** Uninfected BALB/c mice were treated with 500 µg IC, 10F3 or 10G7 or i.p. as indicated. One day later the indicated transcripts in the livers were determined by RT-qPCR. Data correspond to 5 mice/group.
(TIF)

Figure S3 **Increased absolute numbers of leukocytes and CD8+ T cells but not NK cells or CD4+ T cells in the livers of infected mice is not affected by mAb treatment.** BALB/c mice were infected with 100 PFU ECTV in the footpad and treated with the indicated mAbs at 5 dpi. At 2 dpt the leukocytes infiltrating the liver were isolated, counted, stained with various Abs and analyzed by flow cytometry. Graphs indicate the absolute numbers of the indicated leukocytes. Experiment corresponds to five mice/group and is representative of two similar experiments. Statistical analysis using one tailed Mann-Whitney U test showed significant increases in total leukocytes and CD8^+^ T cells (P = 0.0286) in all groups of infected mice as compared to uninfected mice. All other comparisons were not significant.
(TIF)
