# Supplementary figures and images for: Antibody Inhibition of a Viral Type 1 Interferon Decoy Receptor Cures a Viral Disease by Restoring Interferon Signaling in the Liver
Source: PLoS Pathog. 2012 Jan 5;8(1):e1002475. doi: 10.1371/journal.ppat.1002475 (PMC3252373; doi:10.1371/journal.ppat.1002475)

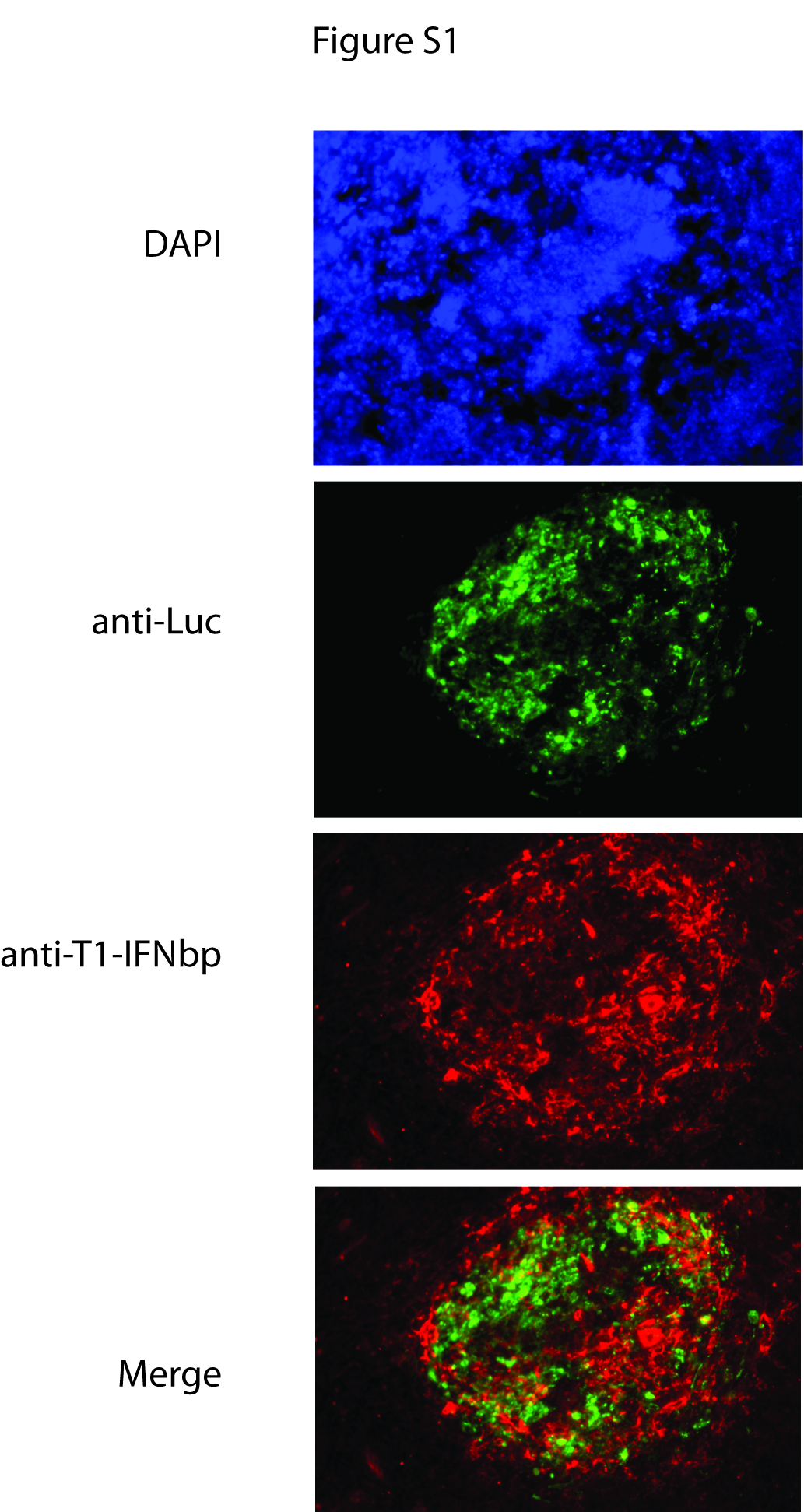

Supplement: Figure S1 — T1-IFNbp binds to infected and uninfected cells in the spleen. A) BALB/c mice were infected with 100 PFU ECTV-Luc. At 5 dpi spleens were harvested and frozen sections stained with anti-Luc Ab to identify infected cells (green) and anti-T1-IFNbp (red). Data are representative of 3 mice and two independent experiments (the original magnification was 200X). (TIF) [file ppat.1002475.s001.tif]

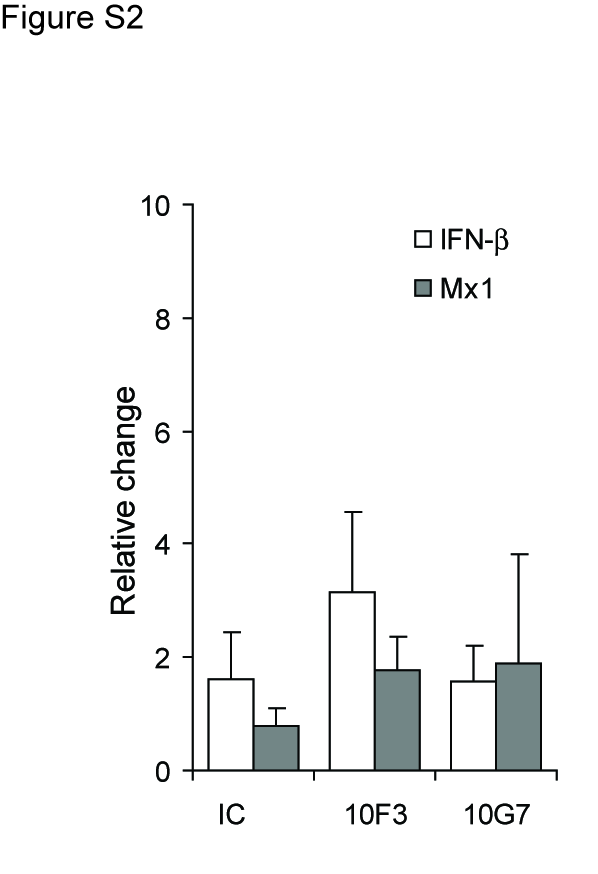

Supplement: Figure S2 — The T1-IFNbp mAbs do not activate IFNAR signaling directly. Uninfected BALB/c mice were treated with 500 µg IC, 10F3 or 10G7 or i.p. as indicated. One day later the indicated transcripts in the livers were determined by RT-qPCR. Data correspond to 5 mice/group. (TIF) [file ppat.1002475.s002.tif]

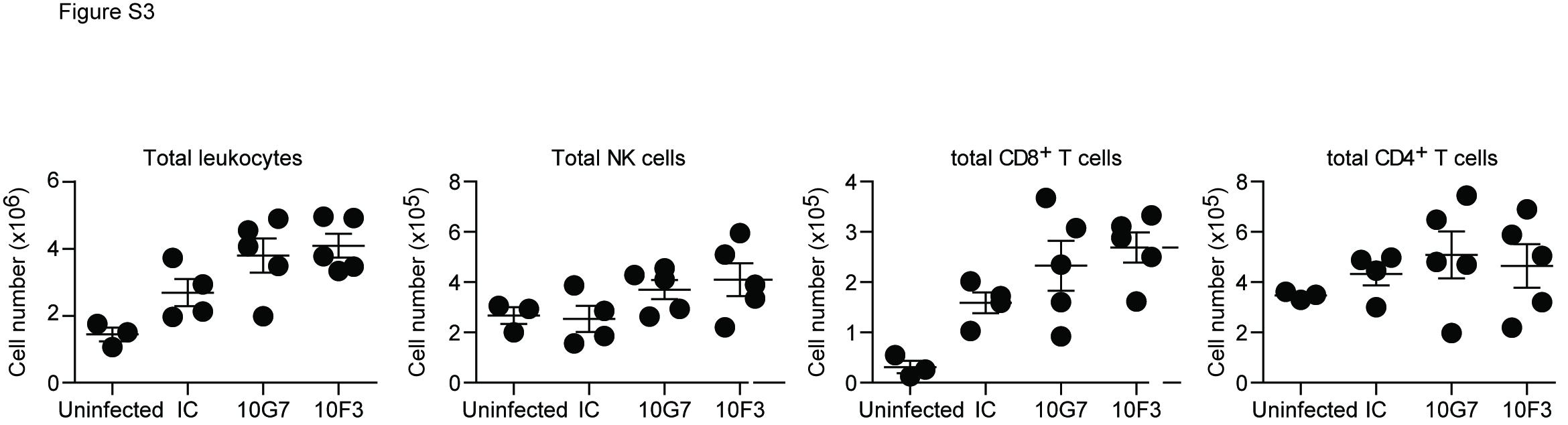

Supplement: Figure S3 — Increased absolute numbers of leukocytes and CD8+ T cells but not NK cells or CD4+ T cells in the livers of infected mice is not affected by mAb treatment. BALB/c mice were infected with 100 PFU ECTV in the footpad and treated with the indicated mAbs at 5 dpi. At 2 dpt the leukocytes infiltrating the liver were isolated, counted, stained with various Abs and analyzed by flow cytometry. Graphs indicate the absolute numbers of the indicated leukocytes. Experiment corresponds to five mice/group and is representative of two similar experiments. Statistical analysis using one tailed Mann-Whitney U test showed significant increases in total leukocytes and CD8+ T cells (P = 0.0286) in all groups of infected mice as compared to uninfected mice. All other comparisons were not significant. (TIF) [file ppat.1002475.s003.tif]
